# Effect of Different Irrigant Activation Techniques on the Penetration of Calcium Hydroxide, an Intracanal Medicament: An In Vitro Study

**DOI:** 10.7759/cureus.48768

**Published:** 2023-11-13

**Authors:** Radha Kalyani Narla, Ravi Kumar J, Tejosmita Chowdary Pavuluri, Krishna Chaitanya P, Ramesh Penumaka, Ratna Kamal Nagelli

**Affiliations:** 1 Conservative Dentistry and Endodontics, Drs. Sudha and Nageswara Rao Siddhartha Institute of Dental Sciences, Vijayawada, IND

**Keywords:** endodontic file, calcium hydroxide, sodium hypochlorite, root canal bacteria elimination, endodontic treatment, sem, irrigant, endoactivator, ultrasonic activation, confocal

## Abstract

Background and objective

The main goal of root canal treatment is to eliminate microorganisms from the canal and to prevent re-infection. To achieve these goals, instrumentation must be combined with adequate irrigation and the placement of intracanal medicament. This study aims to compare the effect of different irrigation activation techniques, i.e., passive ultrasonic irrigation (PUI), EndoActivator (EA), and conventional needle irrigation on the penetration of calcium hydroxide (CH), an intracanal medicament into dentinal tubules.

Methodology

A total of 60 single-rooted extracted human teeth were selected, which were de-coronated to standardize the root length of 12 mm. An access cavity was prepared, and biomechanical preparation was done. The samples were randomly assigned to three experimental groups: Group I received conventional needle irrigation, Group II underwent EA irrigation, and Group III was subjected to PUI. All the samples were filled with CH paste mixed with Rhodamine B dye, and the orifice openings were sealed with Cavit. The samples were stored in an incubator for 24 hours and were subsequently sectioned horizontally at the coronal, middle, and apical thirds using a hard tissue microtome. These were observed under a confocal laser scanning microscope to evaluate the depth of medicament penetration. Statistical analysis was performed using IBM SPSS Statistics for Windows, Version 25.0 (IBM Corp., Armonk, NY). The Shapiro-Wilk test was employed to assess normality, while ANOVA and Tukey's post hoc analysis were utilized to determine significance.

Results

It was observed that the depth of penetration of CH into dentinal tubules was highest in Group III (PUI), followed by Group II (EA irrigation), with the least penetration observed in Group I (conventional needle irrigation). All the groups showed maximum penetration at the coronal third followed by the middle and apical third.

Conclusions

Passive ultrasonic irrigant activation resulted in more penetration of CH into the dentinal tubules followed by EA irrigant activation.

## Introduction

Endodontic treatment aims to eradicate bacteria and their byproducts from the root canal system, create a favorable environment for healing, and prevent reinfection from achieving long-term success [1,2].

Biomechanical preparation of root canals effectively decreases the number of microorganisms in the root canal system. However, due to anatomical complexities such as lateral canals, isthmuses, and apical deltas, complete disinfection is not possible through instrumentation alone [3]. Siqueira et al. revealed that by using three‑dimensional micro‑computed tomography (CT), approximately 10%-50% of the main root canal surface area remains untouched by instruments [4]. Thus, developing advanced endodontic disinfection strategies is imperative for effectively eliminating bacterial biofilm within root canal walls and dentinal tubules. Intracanal medicament effectively eliminates bacterial biofilm and improves the prognosis of root canal treatment [5].

Calcium hydroxide (CH) is the most commonly used among various intracanal medicaments. It is a highly alkaline substance with a pH of approximately 12.5-12.8. The mechanism of action of CH involves the dissociation of calcium (Ca^2^^+^) and hydroxyl (OH^2 -^) ions. The dissociated ions diffuse through the dentinal tubules [6]. Bacteria in root canals adhere to canal walls and penetrate deep into dentinal tubules to a depth of 200-1,500 µm [7]. The antibacterial materials designed for use in root canals should be capable of adhering to the dentin surface and infiltrating into dentinal tubules and being in direct contact with microorganisms. Residual debris and smear layer are barriers to penetration of intracanal medicaments. Hence, the optimal removal of debris and smear layer should be done during root canal treatment.

Several studies were done to analyze the diffusion capacity of CH pastes with different vehicles. Disinfection and debridement sonic and ultrasonic devices were used to improve the efficacy. EndoActivator (EA) is a sonically driven canal irrigation system with a portable handpiece and three types of disposable polymer tips that are smooth and do not cut dentin. The EA system effectively cleans debris from lateral canals and removes the smear layer.

Passive ultrasonic irrigation (PUI) is a noncutting irrigation method done with ultrasonically activated files and can be used with a continuous or intermittent irrigant flow. In this, the energy is transmitted from an oscillating file to the irrigant in the root canal by ultrasonic waves. It induces acoustic streaming and cavitation of the irrigant.

A confocal microscope is used to see the penetration of intracanal medicaments and root canal sealers into dentinal tubules. The confocal microscope produces thin optical sections (0.5-1.5 µm) of fluorescent specimens up to 50 µm thick. Fluorescent dyes such as sodium fluorescein or Rhodamine B dye can be used to visualize the sections [8]. Hence, this study was undertaken to evaluate the penetration depth of CH into the dentinal tubules using different irrigant activation techniques at different levels of the root canal.

## Materials and methods

The study was conducted after obtaining approval from the Institutional Ethics Committee (IEC), Drs. Sudha and Nageswara Rao Siddhartha Institute of Dental Sciences cited by O. C. No. /IEC/10/2018.

Sixty teeth, extracted for periodontal reasons, were studied. Following extraction, the teeth were cleaned of debris and stored in a 0.5% sodium hypochlorite (NaOCl) solution. Specimens were decoronated to a standard 12 mm root segment length with a rotating diamond disc mounted on a micromotor (Marathon, Hero Dental Products, India) under water coolant. A 10-size K file (Mani Inc, Japan) was passed 1 mm beyond the apical foramen to ensure uniform apical patency for all the samples. The working length was determined simultaneously while creating the apical patency by keeping the tip of the instrument 0.5 mm short of the apex. The glide path for all the samples was created with a 15K file. The instrumentation of all samples was conducted using Protaper universal rotary files (Dentsply). The endodontic motor, Endomate DT (NSK), was configured per the manufacturer's instructions. A total of seven instruments were used, three shaping files (SX, S1, and S2) and four finishing files (F1, F2, F3, and F4).

Experimental groups

The specimens were broadly divided into three experimental groups (*n *= 20) based on the final irrigant activation technique used.

*Group I: Conventional Needle Irrigation* *(n* *= 20)*

After drying the canals with paper points, the root canals were irrigated with 3 mL of 5.25% NaOCl, using a 30-G side-vented needle inserted into the root canal without binding, and it was placed 1 mm short of the working length. The irrigant was activated by moving the needle up and down inside the root canal for 30 seconds. The canals were irrigated with 3 mL of 17% ethylenediaminetetraacetic (EDTA) acid as the final irrigant, as described earlier. In this manner, the activation of the irrigant was performed after instrumenting with F1, F2, F3, and F4. The activation was performed with a total volume of 12 mL of NaOCl and 12 mL of EDTA, with 3 mL of irrigant per cycle.

Group II: EA Irrigation (n = 20)

In this group, the root canals were filled with 3 mL of 5.25% NaOCl after instrumentation with F1. The medium size tip (red, 25.04) of EA was taken, and it was kept 2 mm short of working length. The activation of the irrigant was done in 2-3 mm vertical strokes for 30 seconds in between each instrumentation. The canals were cleaned with 3 mL of saline. Later, the canals were filled with 3 mL of 17% EDTA and activated with EA for 30 seconds. In this manner, the activation of the irrigant was performed in between after instrumenting with F2, F3, and F4.

Group III: Passive Ultrasonic Irrigation (n = 20)

In this group, the root canals were filled with 3 mL of 5.25% NaOCl after instrumentation with F1. Ultrasonic irrigation was performed by using a stainless steel ultrasonic Irrisafe file of size 20, mounted on a Satellac ultrasonic unit. The file was kept 2 mm short of working length loosely without binding to the canal. NaOCl was activated for 30 seconds by using a power setting of five. The canals were cleaned with 3 mL of saline. Later, the canals were filled with 3 mL of 17% EDTA and activated with an ultrasonic unit, as described above. In this manner, the activation of the irrigant was done in between after instrumenting with F2, F3, and F4.

Preparation of the CH paste

CH powder was mixed with saline until creamy consistency was obtained. The powder/liquid ratio of the CH paste was 1:1. This paste was mixed with 0.1% rhodamine B. The canals were dried with paper points, and the prepared paste was placed into the root canals using a size #30 Lentulo spiral. The excess medicament was removed, and the coronal openings of the root canals were sealed with a small cotton pellet and cavity to avoid leakage. The specimens were stored at 37 °Cfor 24 hours to allow the medicament to set.

Sectioning of samples

Each specimen was embedded in a circular self-cure acrylic resin mold. Later, the specimens were sectioned perpendicular to the long axis of the tooth by using a hard tissue microtome. The three 1-mm-thick sections were obtained with microtome at the middle of each segment, i.e., coronal, middle, and apical third. The specimens were mounted on a glass slide and examined using a confocal laser scanning microscope. The specimens were mounted on a glass slide and examined using a confocal laser scanning microscope.

Confocal laser scanning microscope investigation

**Figure 1:**
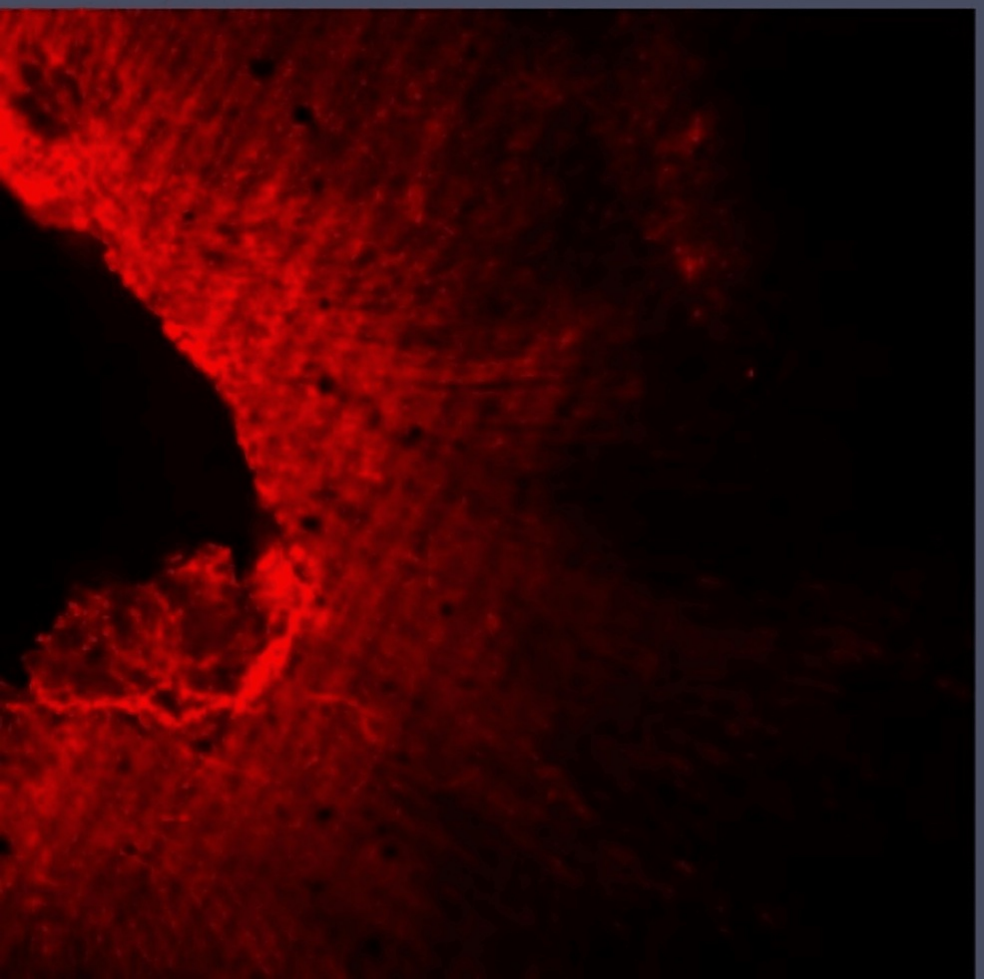
Group I: Confocal laser scanning microscope image of the coronal third region. Confocal laser scanning microscope images of the coronal third showing the depth of penetration of calcium hydroxide medicament with conventional needle irrigation that were scanned from the canal center toward an outward direction under 10x magnification.

**Figure 2:**
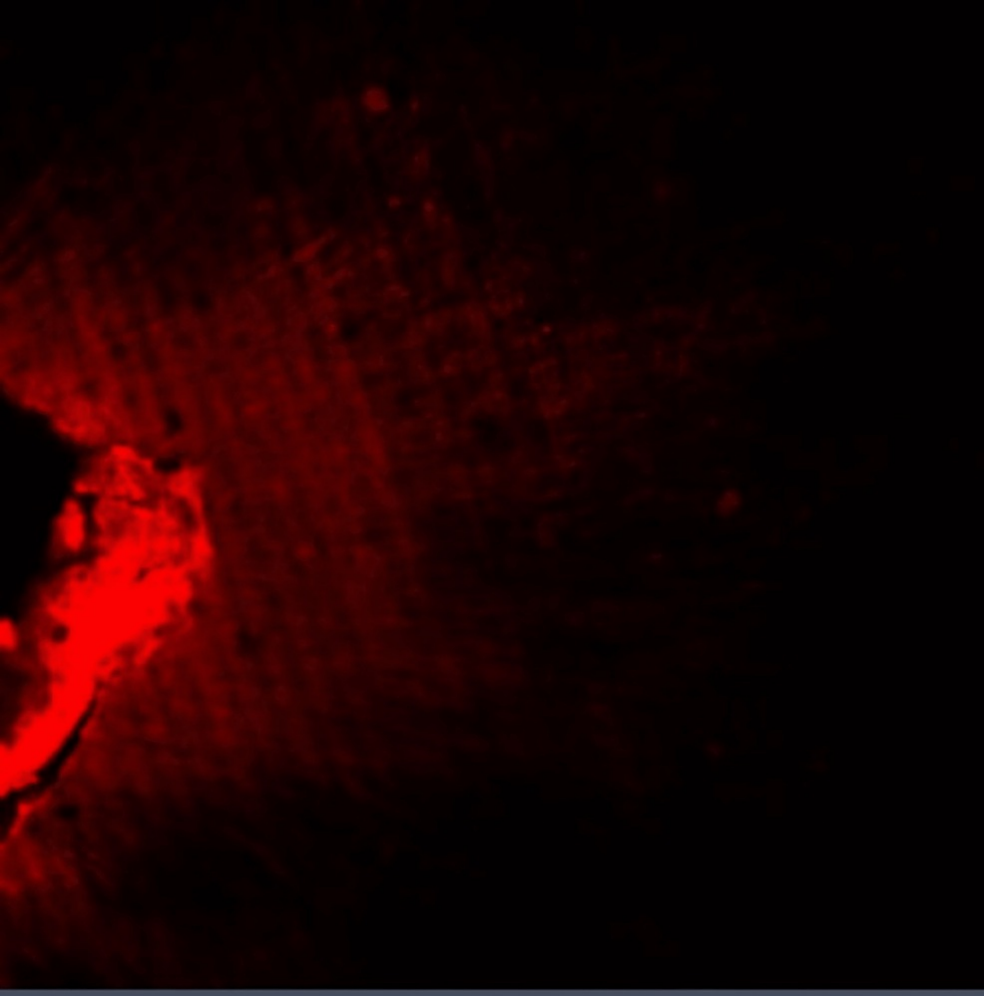
Group I: Confocal laser scanning microscope image of the middle third region. Confocal laser scanning microscope images of the middle third showing the depth of penetration of calcium hydroxide medicament with conventional needle irrigation that were scanned from the canal center toward an outward direction under 10x magnification.

**Figure 3:**
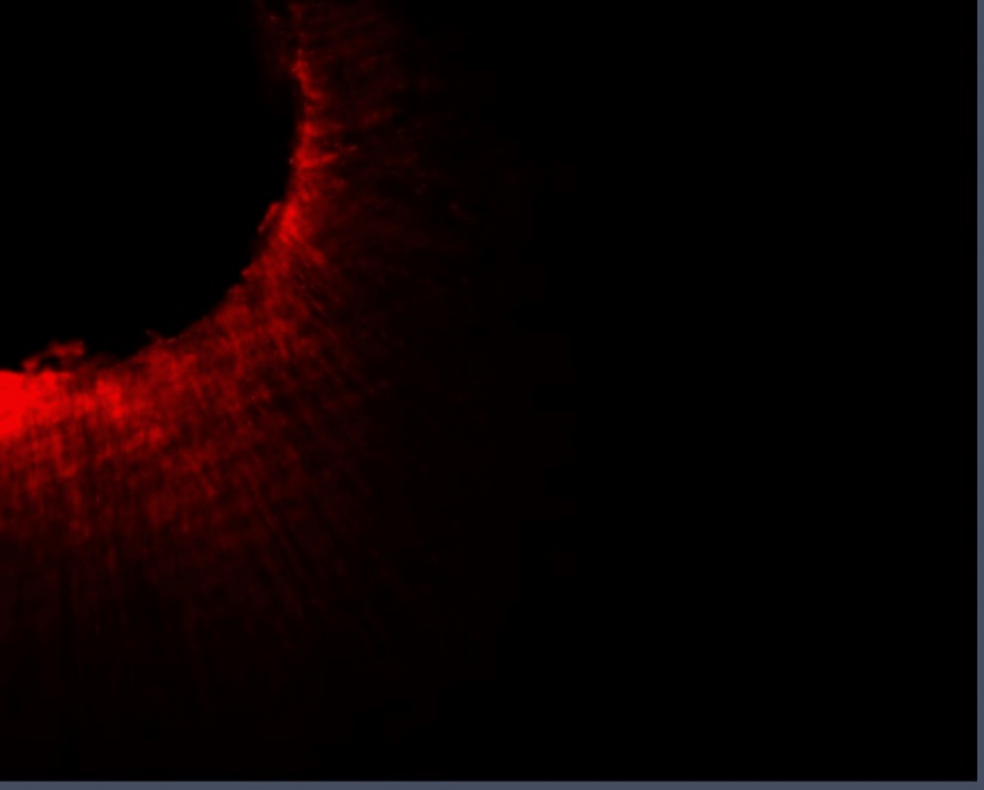
Group I: Confocal laser scanning microscope image of the apical third region. Confocal laser scanning microscope images of the apical third showing the depth of penetration of calcium hydroxide medicament with conventional needle irrigation that were scanned from the canal center toward an outward direction under 10x magnification.

**Figure 4:**
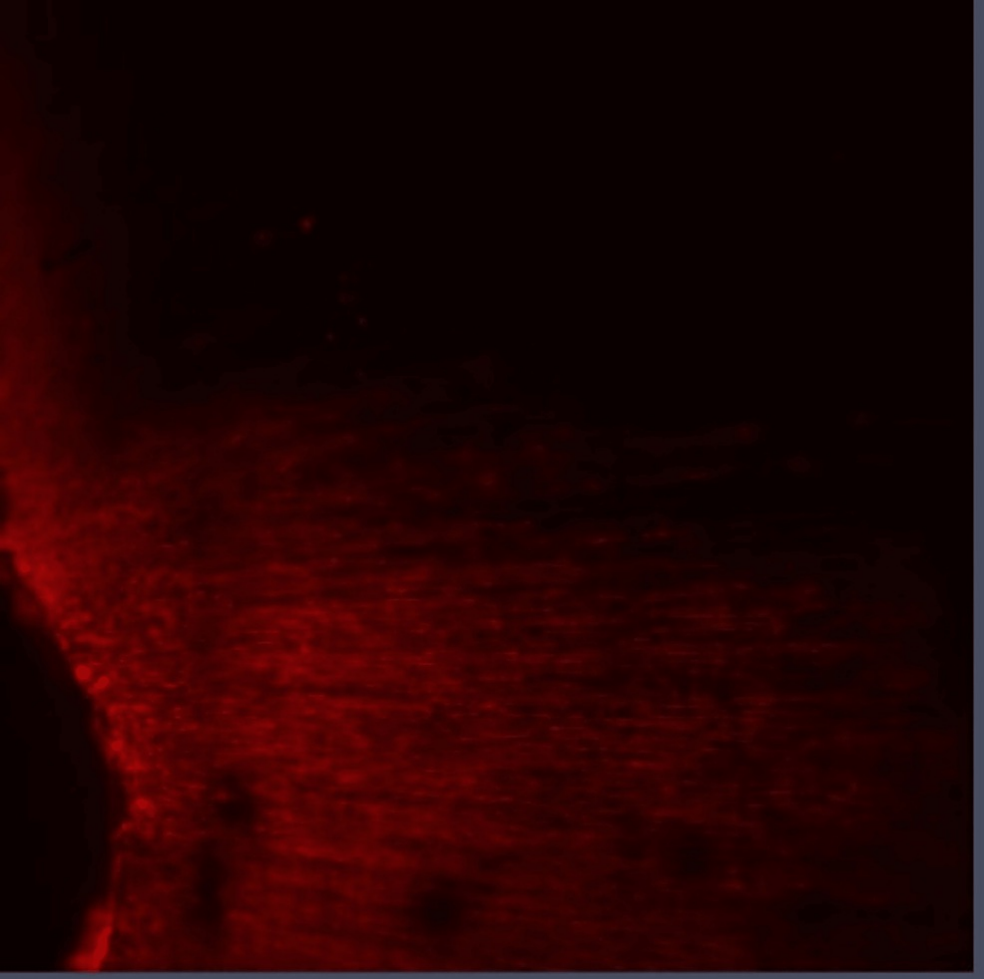
Group II: Confocal laser scanning microscope image of the coronal third region. Confocal laser scanning microscope images of the coronal third showing the depth of penetration of calcium hydroxide medicament with EndoActivator activation that were scanned from the canal center toward an outward direction under 10x magnification.

**Figure 5:**
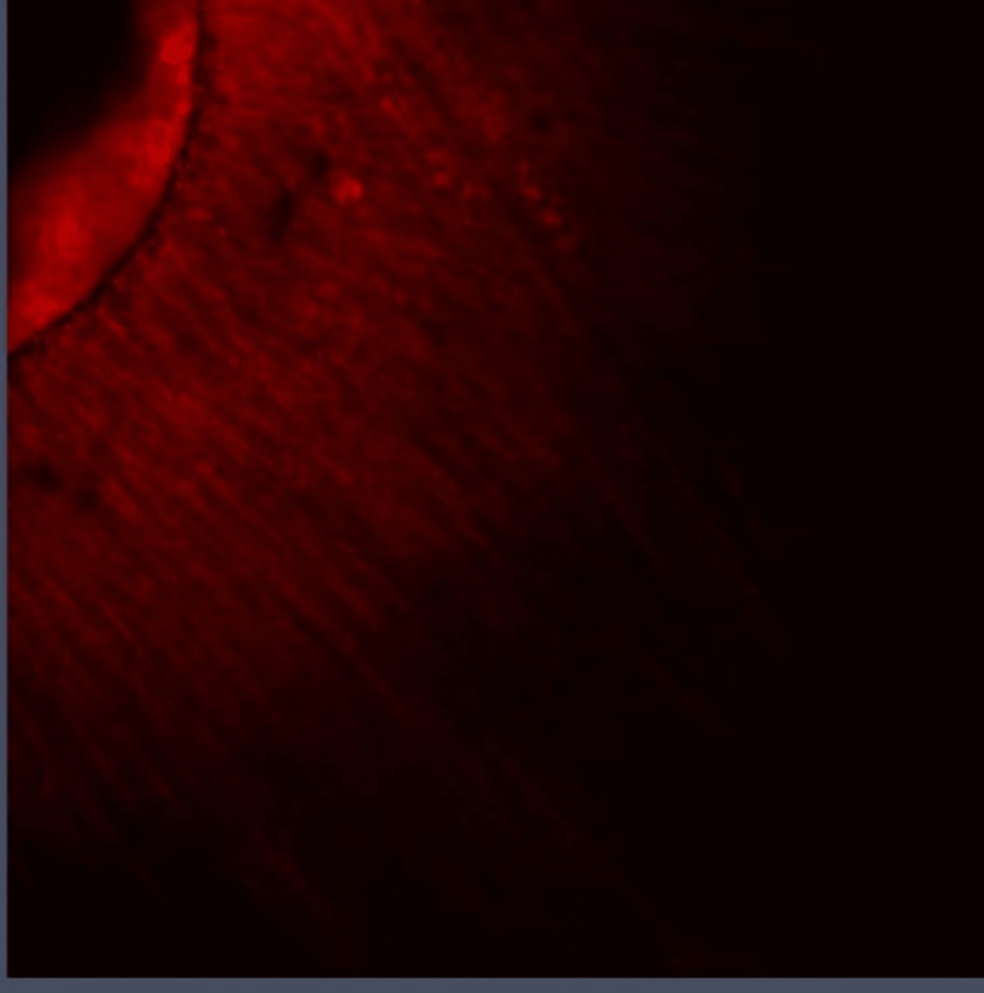
Group II: Confocal laser scanning microscope image of the middle third region. Confocal laser scanning microscope images of the middle third showing the depth of penetration of calcium hydroxide medicament with EndoActivator activation that were scanned from the canal center toward an outward direction under 10x magnification.

**Figure 6:**
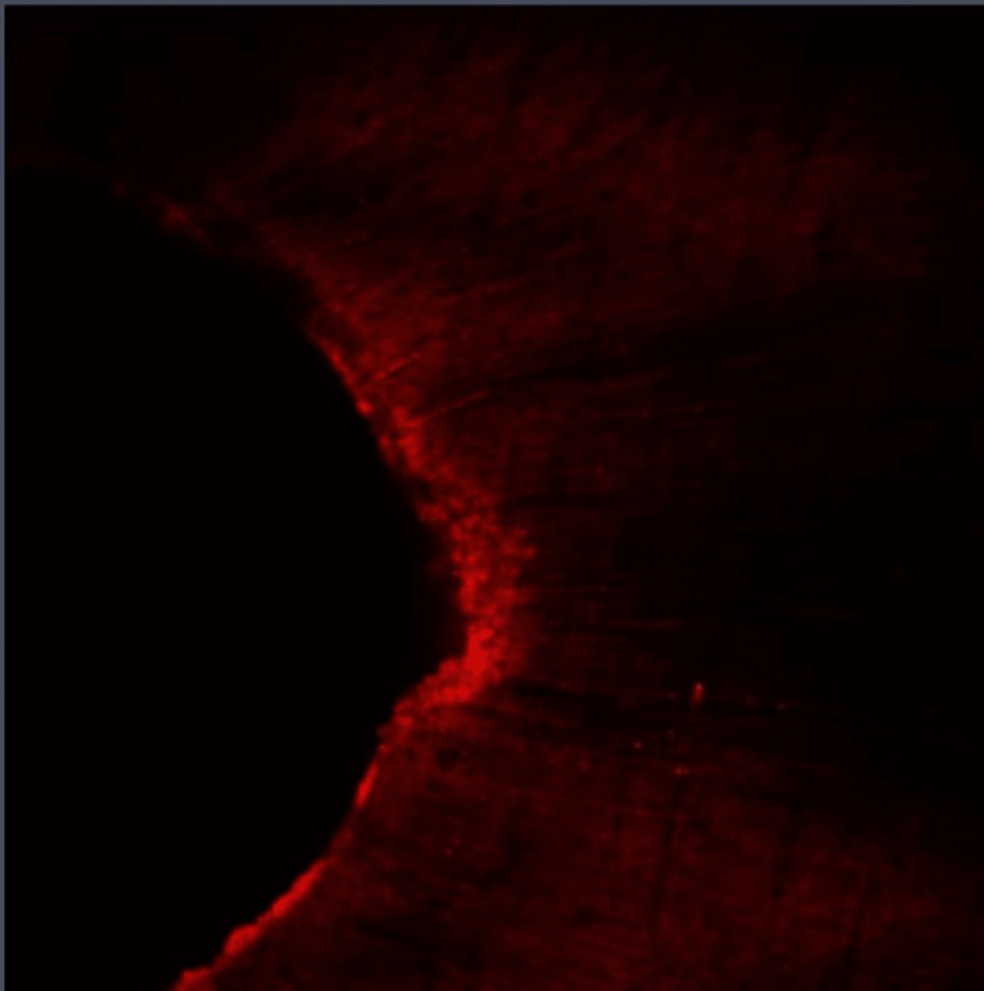
Group II: Confocal laser scanning microscope image of the apical third region. Confocal laser scanning microscope images of the apical third showing the depth of penetration of calcium hydroxide medicament with EndoActivator activation that were scanned from the canal center toward an outward direction under 10x magnification.

**Figure 7:**
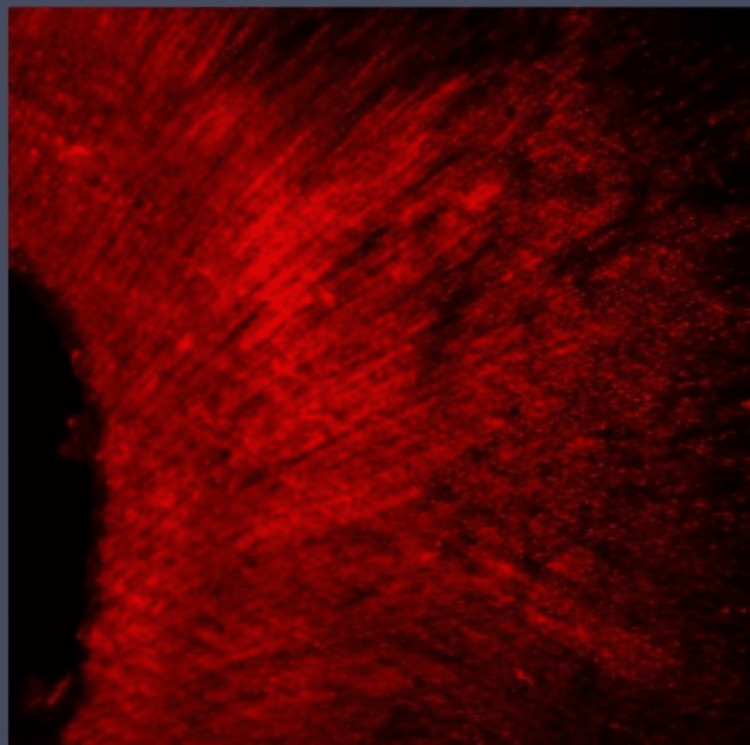
Group III: Confocal laser scanning microscope image of coronal third region. Confocal laser scanning microscope images of the coronal third showing the depth of penetration of calcium hydroxide medicament with passive ultrasonic irrigant activation that were scanned from the canal center toward an outward direction under 10x magnification.

**Figure 8:**
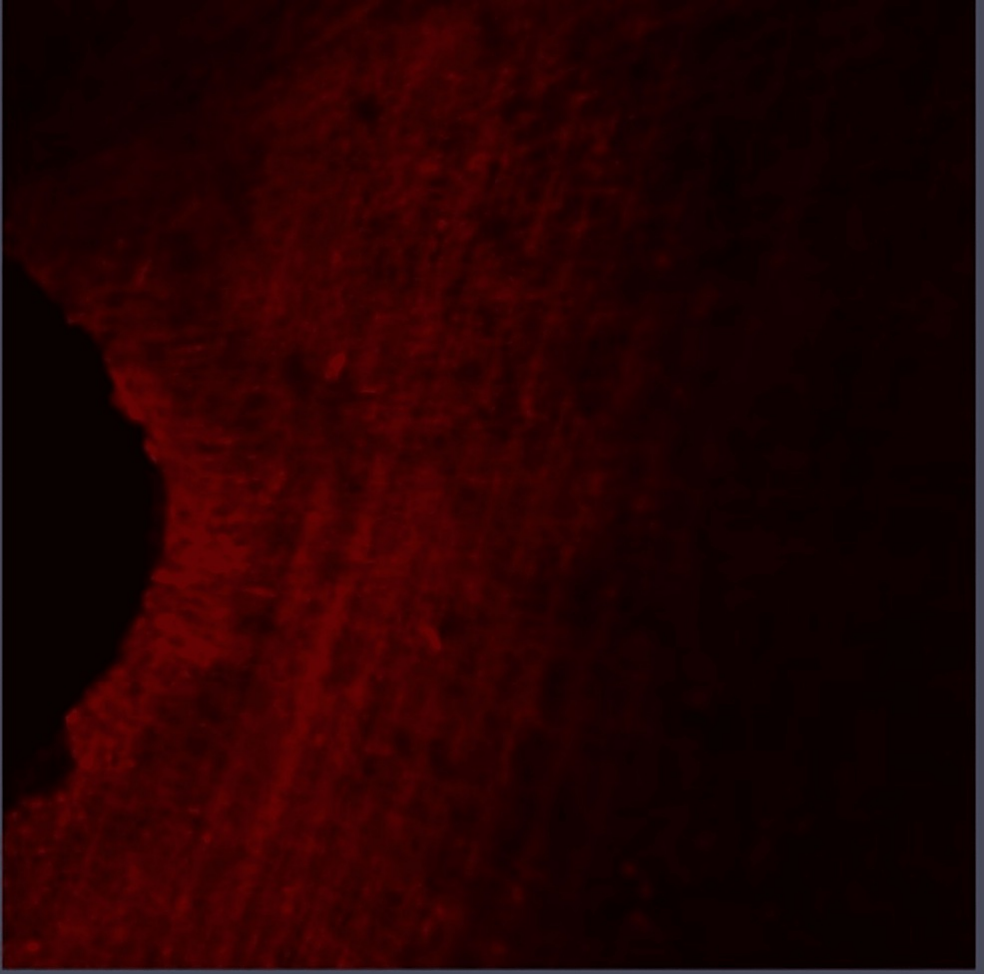
Group III: Confocal laser scanning microscope image of the middle third region. Confocal laser scanning microscope images of the middle third showing the depth of penetration of calcium hydroxide medicament with passive ultrasonic irrigant activation that were scanned from the canal center toward an outward direction under 10x magnification.

**Figure 9:**
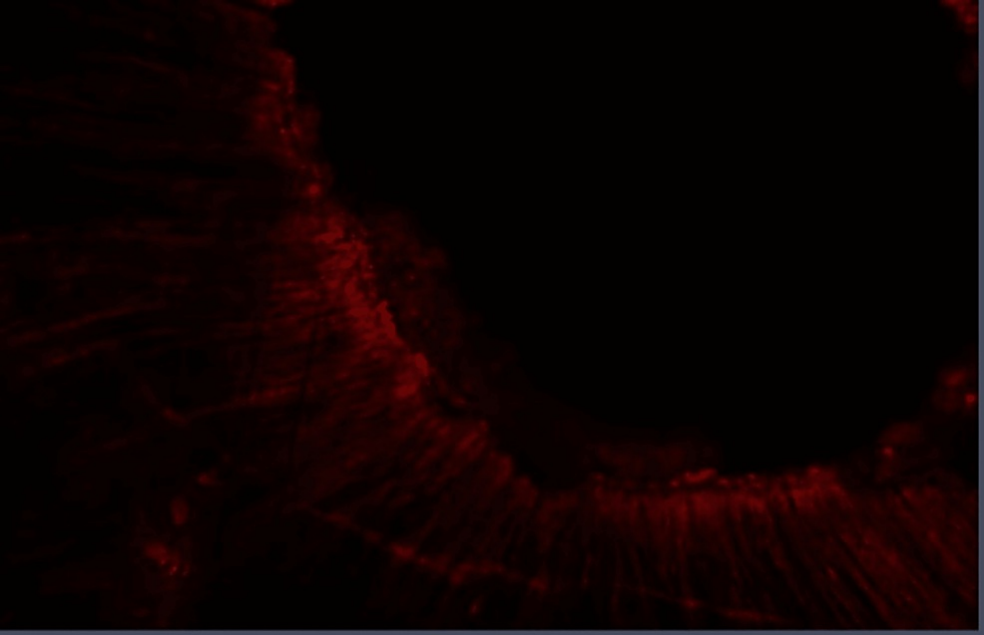
Group III: Confocal laser scanning microscope image of the apical third region. Confocal laser scanning microscope images of the apical third showing the depth of penetration of calcium hydroxide medicament with passive ultrasonic irrigant activation that were scanned from the canal center toward an outward direction under 10x magnification.

For measuring the depth of penetration, the obtained image was divided into five equal parts, and the depth of penetration was measured at each part (Figures 1-9). The average of five values was recorded as the depth of penetration of the sample. These measurements were done using the digital measuring ruler present in Zeiss Microsystems software (Zen Blue, Carl Zeiss AG, Oberkochen, Germany).

Statistical analysis

Statistical analysis was performed using IBM SPSS Statistics for Windows, Version 25.0 (IBM Corp., Armonk, NY). The Shapiro-Wilk test was employed to assess normality, while ANOVA and Tukey's post hoc analysis were utilized to determine significance. A *P*-value less than 0.05 was considered statistically significant.

## Results

**Figure 10:**
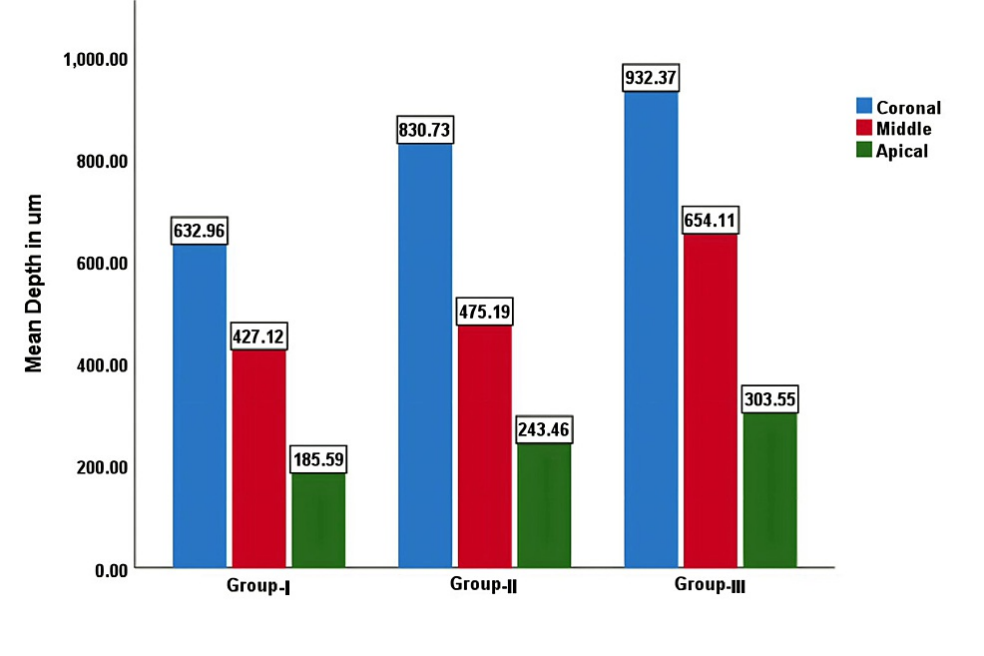
Group I: Bar graph representing the mean values of penetration depths of calcium hydroxide of all groups.

Under the experimental conditions of the current in vitro study, the results showed that irrigant activation techniques increased the penetration of CH paste into dentinal tubules. The highest penetration was seen in Group III (PUI) followed by Group II (EA), and the least penetration was observed in Group I (conventional needle irrigation) (Figure 10).

Intergroup comparison

**Figure 11:**
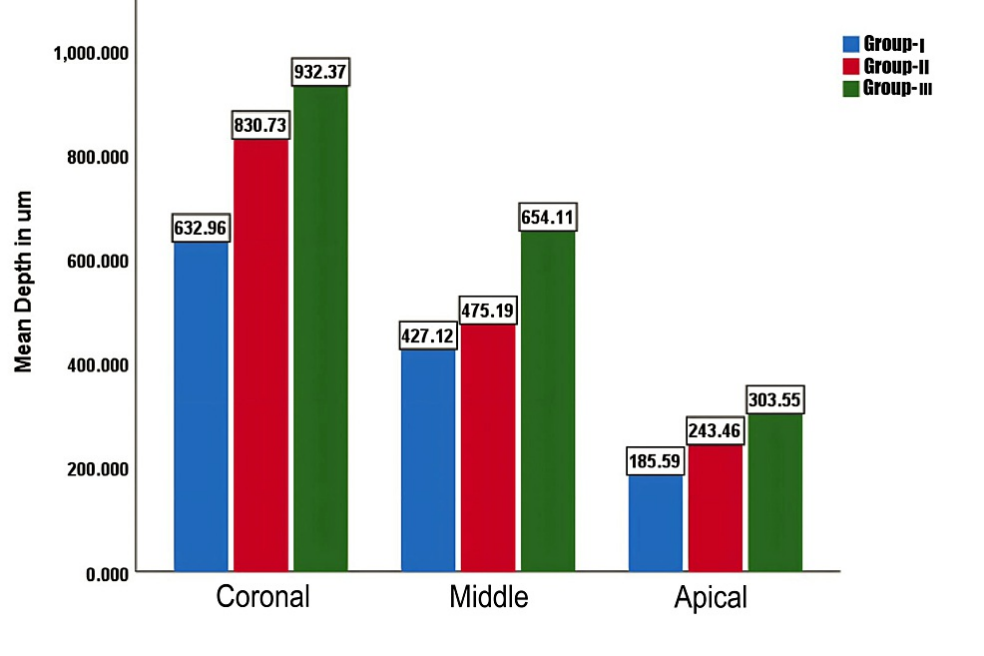
Graph II: Bar graph representing the intergroup comparison of penetration depths of calcium hydroxide of subgroups.

At the coronal third, there was a statistically significant difference between Group I and all the other groups and between Group II and Group I. There was no statistically significant difference between Group II and Group III (Table 1 and Figure 11).

**Table 1:** Intergroup comparision. The aforementioned table describes the intergroup comparison of the mean, SD, and *P*-value of the depth of penetration of calcium hydroxide paste into the dentinal tubules. SD, standard deviation

Depth (µm)							*P*-value
Group	Subgroup	n	Minimum	Maximum	Mean	SD	
Group I	Coronal	20	442.91	866.93	632.96	119.35	<0.001
	Middle	20	213.18	611.63	427.12	102.97	
	Apical	20	84.42	316.42	185.59	74.80	
Group II	Coronal	20	675.61	1030.59	830.73	122.95	<0.001
	Middle	20	315.55	560.12	475.19	65.34	
	Apical	20	62.68	395.54	243.46	105.21	
Group III	Coronal	20	749.40	1184.80	932.37	129.72	<0.001
	Middle	20	414.16	893.51	654.11	148.16	
	Apical	20	132.17	385.61	303.55	65.70	

In the middle third, there was a statistically significant difference between Group I and Group III and between Group II and Group III. No statistically significant difference was observed between Group I and Group II (Table 1 and Figure 11).

In the apical third, there was a statistically significant difference between Group I and Group III. No statistically significant difference was observed between Group I and Group II and Group II and Group III (Table 1 and Figure 11).

## Discussion

The success of root canal treatment depends on accurate chemomechanical disinfection to eliminate pulp tissue, remnants of dentin, and microorganisms. This process effectively eliminates the etiological factors responsible for endodontic infection. The anatomical complexities of the root canal tend to protect the bacterial biofilm from root canal disinfectants and instrumentation procedures, resulting in the progression of endodontic infection. Complete bacterial elimination through instrumentation alone is not possible [9].

Single-visit endodontics cannot be done in all cases. It depends on the severity of the periapical lesion and staging of apical periodontitis. In cases where the lesion size increases by more than 5 mm and a sinus tract opening is present, the placement of intracanal medicament becomes essential. This step is crucial for removing the biofilm and contributing to the long-term success of root canal treatment.

In general, any disinfection strategy in healthcare aims to reduce the bacterial load to a lower level so that the patient’s immune response will allow healing. Studies revealed more postoperative pain in the first week when single-visit root canal treatments were performed when compared to multi-visit root canal treatments. In cases where complex canal morphology is present, particularly in patients with limited mouth opening that prevents completing the procedure in a single visit, the use of CH as an intracanal medicament proves beneficial. The presence of microbial biofilms in the root canal is highly resistant to disinfecting agents, and in such cases, the use of CH in between appointments helps in decreasing the microbial load.

The high pH of CH may not be maintained in the dentinal tubules because of the buffering effect of the dentin, allowing bacterial proliferation. Therefore, an intratubular medicament with maximum penetration would be ideal [10].

Currently, many studies have been carried out to evaluate the efficacy of irrigant activation techniques for canal disinfection, in the removal of intracanal medicaments, and for sealer penetration. In this study, the effect of irrigant activation with an EA and PUI on the penetration of CH was analyzed.

Among various intracanal medicaments available, CH has been widely used in endodontics as an intracanal medicament to eliminate the remaining bacteria after chemomechanical preparation. In this study, a confocal laser scanning microscope was used because it is more advantageous when compared to the remaining techniques. Due to the tortuous pathway of the dentinal tubules, they are difficult to observe in full through a single cut in scanning electron microscopy (SEM). With confocal laser scanning microscopy (CLSM), the sample can exhibit not only on the surface but also in-depth, making multiple cuts and obtaining a three-dimensional image. In this study, Rhodamine B fluorescent dye was used to determine the depth of penetration of the paste in the dentinal tubules.

The presence of a smear layer and debris prevents the penetration of CH. Irrigants must be brought into direct contact with the entire canal wall surfaces for effective action particularly for the apical portions of small root canals. Hence, different irrigant agitation systems were developed for root canal irrigation.

For the success of root canal treatment, the penetration of irrigating solution deep into dentinal tubules is required, which can be done by various irrigant activation techniques. Recently, advances in sonic irrigation like the use of EA effectively remove the smear layer. Other advances include laser-assisted irrigant activation, ozone-based delivery systems, and photodynamic activation. Generally, it is difficult for the irrigants to penetrate in the apical third of the root. To overcome this limitation of negative pressure, devices were introduced such as the EndoVac system and the Rinsendo system.

This study showed that passive ultrasonic irrigant activation showed the highest penetration of CH into dentinal tubules in all the radicular portions when compared to irrigant activation with side-vented needles and EA. Passive ultrasonic irrigant activation had the highest mean value of penetration at the coronal third (932.37 µm), middle third (654.11 µm), and apical third (303.55 µm), with a *P*-value of <0.001. This could be due to the more effective removal of debris with ultrasonic irrigation compared with sonic activation and the higher driving frequency of ultrasound (30 kHz) in comparison to the sonic device (150 Hz).

These results were obtained from a study conducted by Paragliola et al., which assessed the impact of various root canal irrigant agitation protocols on the penetration of an endodontic irrigant into dentinal tubules. The study found that ultrasonic agitation increased the penetration of the irrigant into dentinal tubules compared to gutta-percha agitation and EA [11]. In their study, Mozo et al. concluded that the ultrasonic activation of irrigation with Irrisafe tips proved to be the most effective procedure for eliminating debris and opening up dentinal tubules, particularly in the apical third [12].

Topçuoglu et al. found that PUI removed debris significantly better than CSI and EA [13]. Koçak et al. concluded that PUI irrigation improved the efficacy of all irrigation solutions in the removal of the smear layer in both the coronal and middle thirds, compared to conventional syringe irrigation, especially when considering the apical third [14].

A study by Rödig et al. compared the efficacy of a sonic device (Vibringe), PUI, and syringe irrigation in the removal of debris from simulated root canal irregularities and concluded that PUI was more effective than the Vibringe system or syringe irrigation in removing debris [15].

The depth of penetration of CH in the EA irrigant activation group at the coronal third was 830.73 µm, the middle third was 475.19 µm, and the apical third was 243.46 µm, with a *P*-value of <0.001.

The depth of penetration was lower compared to the passive ultrasonic irrigant activation group. This difference could be attributed to the lower oscillating frequency of EA, which is 190 Hz, in contrast to ultrasonics, where it is 30 kHz. In general, a higher frequency results in a higher flow velocity. The driving frequency also determines the oscillation pattern and amplitude of the instrument, which influences the hydrodynamic effect. The oscillation amplitude of the EA is 1.2 mm and that of the ultrasonic irrigant activation is 0.075 mm. Therefore, the EA will undergo much more wall contact, which inhibits free oscillation of the tip and may reduce the efficient streaming of the irrigant [16]. This could result in lesser debris and smear layer removal from root canals, thus inhibiting the penetration of CH into dentinal tubules. 

The results of Group II in this study were derived from research conducted by Swimberghe et al. In their study, the authors assessed the efficacy of sonically, ultrasonically, and laser-activated irrigation in removing a biofilm-mimicking hydrogel from the isthmus in a root canal model. The findings indicated that ultrasonically activated irrigation outperformed EA [17].

In a study by Sabins et al., the authors evaluated the efficacy of ultrasonic and sonic irrigation on debris removal in maxillary molars and found that PUI produced significantly cleaner canals than passive sonic irrigation [18]. However, contrasting results were obtained in the study by Generali et al. in which they compared the effect of conventional endodontic needle irrigation with EA, Irrisafe, Self-Adjusting File, and EndoVac and found that the use of EA, Irrisafe, Self-Adjusting File, or EndoVac systems in round-shaped straight root canals does not improve sealer penetration into dentinal tubules concerning conventional endodontic needle irrigation [19]. Khalap et al. found that sonic irrigant activation by EA was more effective in smear layer and debris removal when compared with ultrasonics [20].

In this study, side-vented needles were used for irrigant activation in Group I. The depth of penetration in the coronal third was 632.96 µm, the middle third was 427.12 µm, and the apical third was 185.59 µm.

In this study, the depth of penetration was least in the needle irrigation group when compared to the EA and PUI groups. This could be due to lesser penetration of irrigant and lesser debris and smear layer removal, thus inhibiting the penetration of CH into the dentinal tubules.

Results of this study concluded that the maximum penetration of CH in all groups was seen in the coronal third, followed by the middle third, and was least in the apical third. The apical radicular dentin displays less tubule density, with some areas completely devoid of tubules. The effectiveness of smear layer removal techniques is also reduced closer to the apex. The apical radicular dentin shows sclerosis of dentin, preventing deeper penetration of irrigating solutions and root canal sealers. As the diameter of the root canal gradually decreased from the coronal to the apical third, the volume of the irrigant decreased, which decreased the liquid backflow. Thus, less irrigant will be flushed into the apical third than the middle and coronal thirds.

The density of dentinal tubules per square millimeter varies from 4,900 to 90,000. This density of the tubules increases in an apical-coronal direction to the root surface and similarly in an external-to-internal direction from the root surface.

Limitations of this study include that it is an in vitro study and accurate replication of clinical conditions was not achieved. Only single-rooted teeth were analyzed; the results obtained could vary when the same procedures were followed in multi-rooted teeth. In this study, the EA and passive ultrasonic activation techniques were selected and saline was used for making the CH paste. More Intracanal medicaments and other irrigant activation systems could have been used, thus stating that the penetration may vary when different vehicles were used. There is limited literature on the agitation of CH, and additional in vivo studies are necessary to evaluate the long-term impact of CH penetration into dentinal tubules on the prognosis of root canal treatment.

## Conclusions

The present study led to the following conclusions: A statistically significant difference was observed among all groups regarding the penetration of CH into dentinal tubules. Passive ultrasonic irrigant activation demonstrated the highest penetration of CH into dentinal tubules, followed by EA irrigant activation.

Irrigant activation with conventional side-vented needles resulted in less penetration of CH into the dentinal tubules. The maximum penetration of the CH was highest in the coronal third, followed by the middle third, and was least in the apical third.
